# *Drosophila* Trachea as a Novel Model of COPD

**DOI:** 10.3390/ijms222312730

**Published:** 2021-11-25

**Authors:** Aaron Scholl, Istri Ndoja, Lan Jiang

**Affiliations:** Department of Biological Sciences, Oakland University, Rochester, MI 48309, USA; ajscholl@oakland.edu (A.S.); indoja@oakland.edu (I.N.)

**Keywords:** COPD, *Drosophila*, trachea, model

## Abstract

COPD, a chronic obstructive pulmonary disease, is one of the leading causes of death worldwide. Clinical studies and research in rodent models demonstrated that failure of repair mechanisms to cope with increased ROS and inflammation in the lung leads to COPD. Despite this progress, the molecular mechanisms underlying the development of COPD remain poorly understood, resulting in a lack of effective treatments. Thus, an informative, simple model is highly valued and desired. Recently, the cigarette smoke-induced *Drosophila* COPD model showed a complex set of pathological phenotypes that resemble those seen in human COPD patients. The *Drosophila* trachea has been used as a premier model to reveal the mechanisms of tube morphogenesis. The association of these mechanisms to structural changes in COPD can be analyzed by using *Drosophila* trachea. Additionally, the timeline of structural damage, ROS, and inflammation can be studied in live organisms using fluorescently-tagged proteins. The related function of human COPD genes identified by GWAS can be screened using respective fly homologs. Finally, the *Drosophila* trachea can be used as a high-throughput drug screening platform to identify novel treatments for COPD. Therefore, *Drosophila* trachea is an excellent model that is complementary to rodent COPD models.

## 1. Urgent Need to Reveal Novel Mechanisms of COPD

Chronic obstructive pulmonary disease (COPD) diminishes lung function and causes breathing difficulty and is one of the leading causes of death in the United States and worldwide [1]. Various natural and anthropogenic sources of chemical products (e.g., cigarette smoking, wood combustion, vehicle pollution, dust) are the causes of different types of air pollution. As the location of gas exchange, the respiratory system is most susceptible to air pollutants as it is directly exposed to atmospheric toxins. In the past few decades, the incidence of respiratory diseases, such as COPD, has sharply increased [2]. For example, long term exposure to pollutants such as NO_2_, SO_2_, and ozone (O_3_) leads to COPD [3,4,5]. Even exposure to low-level air pollution, when particulate matter (PM2.5), nitrogen dioxide (NO_2_), and black carbon (BC) are below current EU and US limits, has been linked to the development of COPD [3]. Clinically, COPD is characterized by progressive airflow limitations and the development of emphysema, defined by the loss of parenchymal lung tissue [6]. Thus, the loss of surface area for gas exchange in functional alveolar structures is a major hallmark of COPD. According to the Global Disease Burden Study of 2017, an estimated 272 million people worldwide are afflicted with COPD. In 2040, it is expected to be the fourth leading cause of death [1,7].

For the past 30 years, clinical studies and research in cigarette smoke (CS)-induced mouse models consistently showed that reactive oxygen species (ROS)-induced (e.g., O_2_•−, H_2_O_2_, NO) oxidative stress is a significant driver of COPD [8]. In COPD patients, the ROS derives from CS per se and/or is triggered by inflammatory and immune stimuli in epithelial cells of the airways [9,10]. The robust production of intracellular ROS is likely due to defects in oxidative phosphorylation caused by mitochondrial fragmentation due to CS exposure [11]. Incremented oxidative stress further enhances pulmonary inflammation with increased production of inflammatory mediators such as TNFα and Interleukins [9,12,13]. A vicious cycle of persistent inflammation, accompanied by chronic oxidative stress, leads to tissue damage and the progression of COPD [6,14]. Although treating inflammation and ROS is helpful, lung damage remains and continues to be irreversible [15].

The airway epithelium is the first line of defense against pollutants that enter the airways, yet this epithelium must maintain a barrier that is selectively permeable. A recent study shows that ozone-induced damage in airway epithelium structure occurs before the initiation of the inflammation pathways and the production of intracellular ROS [16]. This damage to the cellular junction is due to reduced junction proteins and the increased assembly of actin cytoskeleton that is linked to the tight junction [17,18]. Therefore, systematic studies on the timeline of early and late structural damage, ROS production, and inflammation will greatly help us to understand the developmental process of COPD. Understanding the mechanisms of these changes could provide an opportunity to develop effective therapeutic options that can prevent disease progression. Therefore, a fast, flexible, genetically tractable model organism is crucial to reveal novel mechanisms of COPD that will greatly enhance our understanding of COPD, thereby facilitating the discovery and development of novel and effective treatments.

The *Drosophila* respiratory system (i.e., the trachea) is an excellent system that is complementary to studies in mouse models. The *Drosophila* trachea is comparable to the mammalian lung system with many similarities in development and function. Compared to animal models, the fly trachea model has several advantages: (1) The timeline of early structural damage in the airway, production of ROS, and inflammation due to pollutant exposure can be easily observed through fluorescently-tagged reporter lines without invasive procedures. The *Drosophila* trachea provides the opportunity to observe these changes within live organisms; (2) the involved signaling pathways and cellular processes can be manipulated genetically or pharmacologically to analyze their relevance to the development of COPD phenotypes; (3) it has been estimated that over 60% of human genes associated with diseases have fly homologs [19,20]. Human COPD-associated genes that were recently identified through genome-wide association studies (GWAS) can be screened through GWAS in the *Drosophila* model for their association to COPD; (4) *Drosophila* have been successfully used as a drug screening platform to identify novel drugs for human diseases. Similarly, the *Drosophila* trachea can be used as a drug screening platform to identify novel treatments for COPD; (5) an increased life span can be used as a readout for the effectiveness of the manipulation, which is not suitable in most animal models. These advantages make *Drosophila* an ideal and indispensable model system to identify novel mechanisms of COPD and to explore innovative treatments for the disease that are not currently suitable for mammalian study. 

## 2. *Drosophila* Trachea as a Model to Reveal Underlying Mechanisms of Tube Morphogenesis

**Figure 1:**
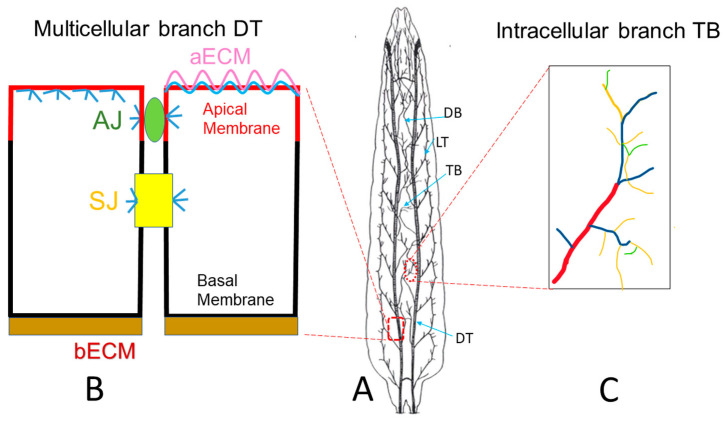
*Drosophila* tracheal branches. (**A**) Schematic larval trachea, an interconnected tubular network. The dorsal trunk (DT) is the multicellular major branch of the trachea. The lateral trunk (LT) and dorsal branch (DB) are unicellular branches. Terminal branches (TB) are intracellular branches, which are formed at the tip of unicellular branches. (**B**) Neighboring cells in DT are connected together by adherens junctions (green AJ) and septate junctions (yellow SJ), the latter of which is equivalent to the tight junction in mammalian systems. Cortical actin (blue) is highly concentrated beneath the apical membrane (red) as well as at the AJ and SJ, and relatively weaker at the basolateral membrane (black). Tracheal cells secrete apical luminal matrix (pink aECM), together with actin (blue), which forms a barrier to protect the apical membrane (red). (**C**). Schematic terminal branches (TB), which are formed in terminal cells, residing at the tip of the unicellular branch such as DB. There are 4 types of TB branches, including Type I (red), Type II (blue), Type III (yellow) and Type IV (green). (**A**) has been adapted from [24].

The *Drosophila* trachea is a ramifying interconnected network of epithelial tubes with a monolayer of tightly-adhered polarized cells surrounding a central lumen [21] (Figure 1A larval trachea). Following the specification of branch identities, fibroblast growth factor receptor (FGFR) signaling guides the migration of tracheal cells in typical directions to form distinct tubes [22]. There are a total of four different types of tubes [23]. Type I multicellular branches are formed by multiple cells connecting together via intracellular junctions, such as the major branch of the trachea, the dorsal trunk (DT). Type II unicellular branches are formed by a linear arrangement of single cells through autocellular junctions, such as the lateral branch (LT) and dorsal branch (DB). Type III tubes are formed by two fusion cells of the adjacent tracheal metameres, with their apical surfaces spanning the inner wall of the ring or donut shape, resulting in seamless tubes without intracellular junctions. Type IV tubes are highly branched intracellular cytoplasmic extensions that form in terminal cells at the tips of the unicellular tubes, such as the branches formed in larval terminal cells. Tube morphogenesis has been extensively studied in Type I multicellular branches such as the DT (Figure 1B) and Type IV larval terminal branches (Figure 1C). In the DT, neighboring cells are connected together by adherens junctions (AJs) and septate junctions (SJs), which are equivalent to tight junctions (TJs) in mammals. SJs control the paracellular barrier function of the trachea while AJs stabilize cell–cell adhesion through the actin cytoskeleton. In *Drosophila*, the SJ is basal to AJ. In addition, cortical actin is highly concentrated beneath the apical membrane as well as the AJ and SJ and to a lesser degree at the basolateral membrane. 

During tracheal development, the tracheal cells secrete material apically to form a transient apical luminal cable at the mid embryonic stage. This apical extracellular matrix (aECM) coordinates apical membrane growth and cell contractility to control tube growth [25,26]. This aECM cable is then degraded and absorbed by tracheal cells [27,28]. From the late embryonic stages through the larval stages, the tracheal cells secrete aECM material to form taenidial ridges that cover the apical membrane. This aECM is soft and flexible to provide ventilation but is also tight enough to function as a protective barrier that shields the tubes from dehydration, infections, and environmental stresses [29,30]

The formation of functional tracheal tubes depends on the sufficient transport of apical proteins, controlled plasma membrane expansion, effective cell junction maintenance, proper connection between the aECM and apical membrane, and coordinated cortical cytoskeleton reorganization. For example, defects in components of the apical secretion pathway such as COPI and COPII complex components Gartenzwerg (garz) and Sec24CD, respectively, lead to the defective secretion of luminal proteins [31,32]; mutations in *lachesin* or *sinuous*, which encode SJ components, lead to the defective secretion of aECM proteins [33,34]; mutations in *dumpy* or *uninflated* (*uif*) disrupt the connection between apical membrane and aECM [35,36]; the formin DAAM regulated actin nucleation and the subsequent polymerization through RhoA is required for actin ring formation, which is critical for the formation of taenidial folds [37,38]; mutations in either *tramtrack* or *grainy head* cause defective apical membrane expansion [39,40]. Taken together, disruption in any of these processes will lead to malformations in the trachea. 

In addition to Type I multicellular tubes, such as the DT, tube morphogenesis has also been extensively studied in Type IV intracellular branches in larval terminal cells. Type IV terminal branches contact target tissues for gas exchange, which is similar to alveoli in human lung. The differentiation of terminal cell involves two processes, branching and subcellular lumen formation. Branching in the terminal cells heavily relies on cell migration. FGFR signaling, likely through the downstream GTPases Rac1 and Rac, allows cytoskeleton remodeling and filopodia formation for terminal cells to start branching [41]. Following the branching morphogenesis, it is essential to form a lumen within these branched structures primarily for the transport of gases. 

Terminal cell branching and subcellular lumen formation are intimately associated. Subcellular lumen formation involves the structured expansion of the apical plasma membrane through the dynamic modulation of vesicle transport, which depends on the reorganization of the cytoskeleton. The initial subcellular lumen develops by invagination of the apical membrane inside the cell. The ingrowing apical membrane accumulates apical markers such as the PAR-polarity complex components aPKC/Par6/-Baz and Crumbs (Crb) [42,43]. Following apical membrane formation, aECM material is accumulated inside the lumen creating the aECM [44]. The growth of the subcellular lumen depends on intracellular trafficking of membrane and lumen material [41]. For example, Rab11 is involved in the trafficking of recycled and newly synthesized proteins to the apical plasma membrane [45]. In addition, other endosomes coordinate plasma membrane redistribution. For example, organelles carrying late endosomes and multivesicular bodies (MVBs) markers act as a transit station to redistribute the membrane apically and basally in terminal cells [46]. Therefore, coordination between cytoskeleton reorganization and vesicular trafficking is critical for terminal branch formation.

Unlike the embryonic tracheal system, which develops in a stereotypical and genetically controlled manner, the development of the larval trachea exhibits plasticity and also adapts to particular oxygen needs of the different tissues of the body. Insufficient oxygen levels activate the hypoxia signaling pathway, which is largely regulated by hypoxia-inducible factor 1α (HIF-1α) [47]. HIF-1α is a transcription factor that is considered to be a master transcriptional regulator of O2 homeostasis [48]. HIF-1α induces the transcription of genes related to angiogenesis, cell proliferation/survival, as well as inflammation [49,50]. The HIF-1α signaling pathway is activated in smokers with COPD. Thus, the increased expression of HIF-1α, VEGF, and VEGF receptor 2 were associated with decreased lung function, reduced quality of life, and progression of COPD [51]. The *Drosophila* functional homolog of HIF-1α is Similar [52]. Hypoxia-induced activation of Similar leads to over branching caused by elevated *Drosophila* FGF, Branchless (Bnl) [53] as well as increased ROS levels, similar to what was observed in COPD patients [54]. 

The *Drosophila* adult tracheal system, called air sacs, forms during the pupal period. The development of the air sacs starts from the third instar larval stage. The air sac precursor cells bud from the larval trachea, proliferate, and migrate towards the wing imaginal disc to form air sac primordia (ASPs). During pupal stages, the cells in ASPs migrate to form branches. Thereafter, they cease migrations and begin to elaborate into air sacs, which will further expand in adults. The air sacs are associated with numerous bundles of trachea, which extensively interdigitate with flight muscle to supply oxygen [55]. The development of ASPs involves three processes: cell proliferation, downregulation of adhesion molecules at tip cells, and extracellular matrix remodeling. 

The morphogenesis of ASP is directed by FGFR signaling-induced outward migration of distal tip cells towards the wing imaginal disc, which secretes the *Drosophila* FGF, Bnl [55,56]. In addition, FGFR signaling induces the expression of epithelial growth factor (EGF)/vein, which activates epithelial growth factor receptor (EGFR) signaling to stimulate cell proliferation and survival [56,57]. The turnover of EGFR and FGFR is mediated by endosomes. For example, compromised endosomal sorting complex leads to impaired ASP development [58]. For the proper cell migration during ASP development, down-regulation of cell adhesion molecules such as *Drosophila* E-cadherin (shotgun), and *Drosophila* β-catenin (armadillo) is required in the tip cells [59]. Furthermore, remodeling of the ECM is necessary to facilitate the growth of ASPs. ASPs arise from a region of a tracheal branch that is directly juxtaposed to the wing disc [55]. This tracheal region lacks a visible tracheal-specific ECM, called basal lamina (BL), which is proteolyzed by the endopeptidase Mmp2 [60,61]. It was reported that elevated BL in *mmp2* mutants leads to stunted ASP growth and failed formation of functional air sacs [61]. Another class of proteases, cathepsins, have also been implicated in ECM remodeling around the ASP [59]. The reduced BL thickness allows for greater FGF signaling response, similar to what is observed during mammalian lung development [62,63]. 

## 3. *Drosophila* Trachea as a COPD Model to Systematically Study the Development of COPD

Long-term CS exposure is by far the most important risk factor for COPD. Thus, chronic CS exposure has been used to investigate the mechanisms of COPD in mouse models [64,65]. Overall, for COPD, the failure of repair mechanisms to cope with the increased ROS and inflammation in the lungs leads to the loss of functional alveolar structure. As such, signaling pathways associated with tissue repair and ROS production have been extensively studied. For example, tissue repair relevant Wnt signaling [11] and JAK/STAT signaling [66], as well as cytokines (IL5 and IL6) that activate the JAK/STAT signaling, have been identified as potential drug targets [66]. In addition, the Nrf2 (NF-E2-related factor 2) pathway is also strongly activated by CS exposure and linked to COPD development [67]. The Nrf2 pathway is a potential drug target due to its function in controlling the expression of antioxidant genes that ultimately exert anti-inflammatory functions [68]. Despite the progress made towards COPD research, the molecular mechanisms underlying the development of COPD are still not well understood. This is reflected by the lack of effective treatments for this disease [15,69]. Thus, informative and druggable animal models are highly valued and desired. A simple model, such as the *Drosophila* trachea, may provide promising new candidates that are complementary to the current knowledge of COPD. 

**Figure 2:**
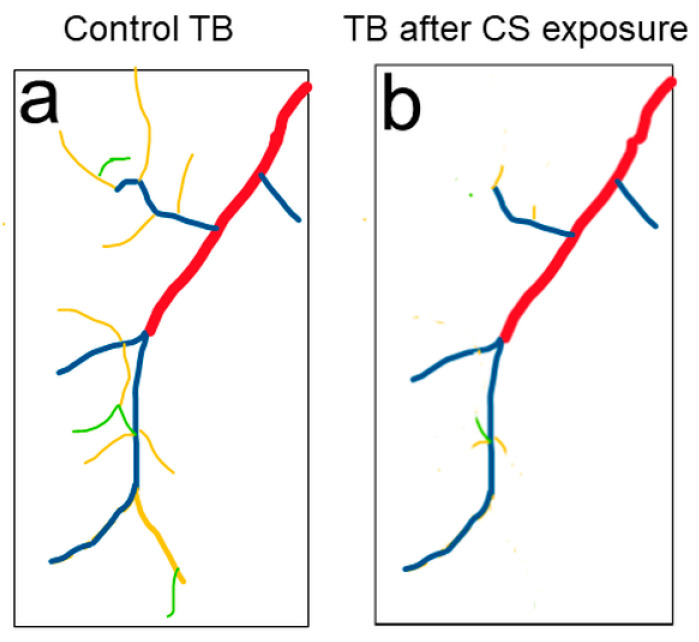
Tracheal morphology changes were observed in 2nd instar larvae upon CS exposure. (**a**) Schematic of a terminal cell of the dorsal branch in the third segment of *Drosophila* 2nd instar larvae. Terminal branches (TB) include Type I (red), Type II (blue), Type III (yellow) and Type IV (green) branches. (**b**) Type III (yellow) and Type IV (green) TBs are significantly reduced upon CS exposure.

CS exposure affects functions in various organs in *Drosophila*. CS exposure has been shown to increase heart rate and cause alterations in the dynamics of the transient increase in intracellular calcium in myocardial cells [70]. CS and nicotine exposure have also been linked to changes in *Drosophila* sexual behavior, larval brain size, and the adult fly dopaminergic system [71,72]. Recently, the CS-induced *Drosophila* COPD model showed a complex set of pathological phenotypes that resemble those seen in human COPD patients [73]. These phenotypes include premature death, reduced physical activity, enhanced metabolic rates, and reduced respiratory surfaces in *Drosophila* trachea. Due to the short life span of fruit flies, the survival rate was measured within 2 weeks, compared to a survival rate of months in the mouse model. The physical activity of a large quantity of flies was automatically evaluated by the *Drosophila* activity monitoring system [74]. Metabolic rate was measured by body fat content using ELISA [75]. The reduced respiratory surface was analyzed in live 2nd instar larval terminal cells by using a btl:GFP transgenic line without an invasive procedure (Figure 2). Similar to the reduced alveolar surface area observed in human COPD lungs, Type III and Type IV branches in CS-induced COPD *Drosophila* larvae show the most obvious COPD phenotype: reduced numbers and overall length [73]. Comparably, as chronic CS exposure in the mouse model leads to the development of COPD, chronic CS exposure also leads to the development of COPD-like phenotypes in *Drosophila* larval trachea. 

The early junctional defects upon pollutant exposure in the mouse model suggest other early structural damages. These damages can be studied in the *Drosophila* larval multicellular DT. Various structures, including the aECM, basal ECM (bECM), apical membrane, basal membrane, junctions, and cytoskeleton in DT, can be easily observed using available transgenic lines with fluorescently-tagged structural proteins in live organisms (Figure 1B). These lines include the aECM (Vermiform:RFP and Serpentine:GFP [76] and bECM (type IV collagen Col41A:GFP [77]), apical membrane (Crumb:RFP and Crumb:GFP [78]), basal membrane (αSpectrin:GFP [79]) actin cytoskeleton (actin:GFP [80], AJ junction (DEcad:GFP, RFP [81]), SJ (Discs Large:GFP [76]). The later structural damage, reduced numbers, and length in terminal branches can be observed in larval trachea using btl:GFP lines [82]. Therefore, the *Drosophila* larval trachea can be used as a COPD model to systematically study the timeline of early- and late-stage structural damage upon chronic pollutant exposure.

In addition to the timeline of structural changes in the *Drosophila* COPD model, their relevance to known molecular mechanisms and components of tube morphology can be tested. For example, the activation of the MAPK/ERK pathway leads to the disruption of the tight junction [83]. The RhoA/ROCK signaling pathway leads to disruption of the tight junction through the aggregation of cytoskeletal actin [84]. Pollutant exposure could also cause structural damages through other mechanisms such as defective apical membrane expansion, aECM formation, or tube size maintenance. Similarly, the involvement of the underlying mechanisms, such as the protein trafficking pathway and cytoskeleton reorganization, in the development of COPD can be further studied in the larval trachea. 

Along with structural damage, the intracellular production of ROS can be indirectly measured by ROS-inducible gstD (glutathione S transferase D)-GFP reporter, gstD-GFP [85]. Overall levels of peroxides can be detected using 2′,7′-dichlorofluorescein (H2DCF) followed by imaging as described [86]. Furthermore, increased concentrations of multiple cytokines, such as TNFα and interleukins, orchestrate chronic inflammation in COPD [12,13,87]. The only *Drosophila* TNF superfamily member is Eiger (Egr) [88,89]. Three cytokine molecules, namely Unpaired (Upd), Upd2 and Upd3, function similarly as interleukins [90,91,92]. Reporter lines *egr-lacZ* and *upd-lacZ* lines are available to measure the expression of these inflammatory cytokines [73]. It is also possible to observe the production of these inflammatory cytokines in vivo by generating fluorescent protein-tagged reporter lines. Previous studies in *Drosophila* trachea have revealed genes, pathways, and cellular processes involved in tube morphogenesis. The relevance of these mechanisms to the development of COPD can be further studied in the *Drosophila* trachea. Thus, the larval trachea provides an excellent model to systematically study the timeline of early and late structural damage, production of ROS, and inflammation upon pollutant exposure in vivo. However, the larval trachea is roughly equivalent to adolescence, while the adult trachea is comparable to adult human lungs. A recent study showed that early COPD in young adults is associated with clinical COPD 10 years later [93]. Therefore, the mechanisms of COPD revealed by studies of *Drosophila* larval trachea would be more relevant to the initiation of COPD in young adults. Although the current knowledge of the *Drosophila* adult trachea is still limited, studies of the development of air sacs provide opportunities to unveil mechanisms of COPD in relation to the disease progression in adult trachea. For example, the morphological changes in the air sac (*btlenhancer-mRFP1moesin* [57]), tracheal BL (collagen IV:GFP and Perlecan:GFP [61]), and junction proteins (Dα-cat-GFP, RFP [81]) can be visualized through fluorescently-tagged structural proteins in the late pupal stage, when air sacs form. 

## 4. *Drosophila* Trachea as a Model to Reveal Novel Mechanisms of COPD

In addition to testing the relevance of previously identified mechanisms of tube morphogenesis to the development of COPD, the *Drosophila* trachea can be used to identify novel mechanisms underlying the development of COPD or to verify the relevance of COPD-associated genes identified by GWAS. RNAseq analysis of CS-exposed COPD fly model identified novel cellular processes and genes associated with COPD. Not surprisingly, RNAseq analysis performed with airway epithelia of CS-exposed and non-treated flies identified changes in genes that are deeply involved in responses towards detoxification of xenobiotics (such as cytochrome P450), ROS (glutathione biology), and inflammation (cytokines) [73]. The abundant presence of these genes in the lungs of smokers is thought to be intimately connected to disease development [6]. In addition, signaling pathways associated with COPD such as Nrf signaling, which mediates antioxidative response and JAK/STAT signaling involved in repair processes, are also deregulated [73].

Many genes involved in other cellular processes such as transmembrane transport (*CG10019*, *CG11897*, *CG13223*, *CG31793*, *Esp*, *MFS14*, *MFS3*, *Mrp4*, *MRP*, *Tpc1*, *outsiders*) and anion transport (*CG15096*, *MFS14*, *MFS3*) have also been identified in relation to COPD in *Drosophila* [73]. For example, the major facilitator superfamily (MFS) is the largest and most diverse superfamily of transporters found in all living organisms [94]. MFS proteins transport a broad spectrum of ions and solutes across membranes via facilitated diffusion, symport, or antiport [95]. MFS transporters are pivotal at the cellular level for growth, metabolism, and homeostasis in all organisms. Defects in MFS transporters are associated with a plethora of serious diseases such as cancers and metabolic diseases [96,97]. Another example is a member of the *slc16* gene family of monocarboxylate transporters, *outsiders,* which encodes a monocarboxylate transporter involved in programmed cell death [98]. The functions of these genes in the development of COPD, however, are poorly understood. Therefore, investigating the role of these genes in a *Drosophila* COPD model will provide novel insights into the development of COPD. 

As interactions with the environment, individual behaviors, and genetics vary widely among individuals across diverse backgrounds, it is crucial to apply statistical approaches to large genetic datasets in order to determine the significance of a genetic variant among a population. GWAS are used to provide evidence for common genetic variants in a population that contribute to the overall pathology of a disease. In each COPD-GWAS, variant data were analyzed by comparing genetic differences in individuals with COPD to their control counterparts without COPD. Several recent GWAS findings have detailed numerous COPD susceptibility genes [99,100,101,102]. Direct *Drosophila* homologs of these COPD-associated genes were determined using the HomoloGene search function through NCBI and are reported in Table 1. The *Drosophila* RNAi Screening Center Integrative Ortholog Prediction Tool (DIOPT; http://www.flyrnai.org/diopt (accessed 26 October 2021)) was used to screen genes reported in previous GWAS for orthologs [103]. Only moderate and high confidence orthologs with DIOPT scores above 2.0 were reported in Table 1. 

A mechanistic investigation of the functions of homologous and orthologous human COPD-associated genes will improve our collective understanding of the roles that these genes play in the development of COPD. In one such case, human fibroblast growth factor 18 (FGF18) is necessary for cell proliferation, differentiation, and migration, while the homolog of FGF18 in *Drosophila*, called B, shares the function of cell migration within the developing trachea [22]. For example, it is required for the branching process of terminal cells to form TB branches [41], which are significantly reduced in the *Drosophila* COPD model [73]. Genes involved in tissue and matrix interactions such as Adam19 and COL15A1 are intriguing as defects in the extracellular matrix, which leads to tube structure damage [35,36]. Moreover, it is not surprising that genes involved in vesicular trafficking, such as Rab4, are associated with COPD, as vesicular trafficking is important for tube morphogenesis [45]. Taken together, elucidating the functions of these genes in the development of COPD in *Drosophila* trachea will greatly help us to understand the function of their human homologs in COPD. 

## 5. *Drosophila* Trachea as a Screening Platform to Identify Novel Treatments for COPD

Although rodent COPD models can recapitulate major features of COPD to study the underlying mechanisms, it is almost impossible to carry out long-term studies, as those utilize life span as a major readout. Moreover, these systems are not suited for high-throughput approaches to evaluate the effectiveness of novel therapeutic interventions. With ongoing research in model organisms, the underlying molecular mechanisms and potential drug targets for COPD will expand significantly in the near future. Furthermore, with the access of both synthetic and natural chemical libraries, there is an urgent need to develop an effective way to screen rich resources for chemicals with the potential to treat COPD. 

Activity-based and phenotype-based screens are two major types of drug screens. Activity-based screens rely on known targets, which are critical to specific diseases such as enzymes or signaling molecules whose activities can be detected. The phenotype-based screens are complementary to activity-based screens, which rely on the rescue of the complex biological phenotypes, such as life span or morphological defects [104]. *Drosophila* have been successfully used for phenotype-based screens, such as the identification of drugs for colorectal cancer (CRC) [105] and for spinal muscular atrophy (SMA) [106] as well as phenotype-based screens to identify a sleep-promoting drug and drugs for Parkinson’s disease [107,108]. 

**Figure 3:**
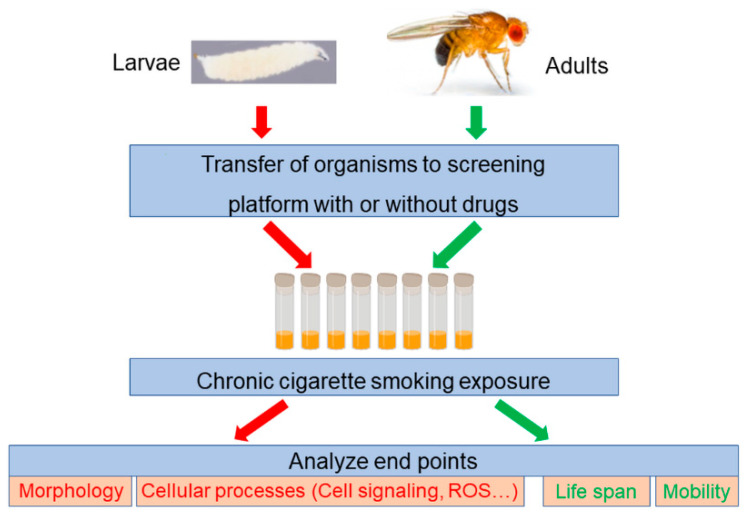
*Drosophila* drug screening platform. Organisms (either adult or larvae) are transferred to vials containing media with or without drugs. These vials are then exposed to CS chronically. Finally, the end points can be analyzed. Larvae can be used to analyze morphology, cellular processes such as cell signaling, ROS, etc. Adults can be used to analyze life, mobility, etc.

The *Drosophila* COPD model has also successfully confirmed drug targets identified by previous research. Impairments in the Nrf2 mediated antioxidative response in the lung is associated with the development of COPD [109]. Manipulation of this signaling pathway with oltipraz, a specific Nrf2 activator, increased median life spans in CS-treated flies by approximately 15% [73]. A drug screen platform can be established to identify novel drugs or combinations of drugs for the treatment of COPD (Figure 3). For example, a phenotype-based screen with life span as a readout can be used to screen chemical libraries for novel treatments of COPD using adult flies. Specifically, 15–20 adult flies (5–7 days old)/per vial are kept on media containing individual drugs or combinations of drugs, with flies kept on standard media being used as controls. These flies are exposed to CS once a day for 30 min, 5 days a week. Two days after the completion of CS-exposure, the survival rate will be analyzed. The drugs that improve survival rate can be considered potential drug candidates for COPD. Additionally, activity-based screens to identify drug treatments that target cellular processes, such as restoring structural damage or reducing ROS, can be carried out in *Drosophila* larval trachea. This is performed by observing fluorescently-tagged structural proteins in vivo without the need for invasive procedures. Since air sacs in adult flies are equivalent to human adult lungs, the effectiveness of potential COPD drugs identified by screens that utilize larval trachea would need to be further tested using adult flies. 

## 6. Summary

Animal models are heavily utilized to reveal current knowledge of lung diseases, such as COPD [23]. However, due to the complexity of these systems, animal models are rarely used to identify potential novel pathways in lung diseases. Thus, the current concepts underlying COPD development and its causes have seen minimal change for three decades. Hence, a tremendous need exists for new breakthroughs underlying the development of COPD using simpler model organisms such as *Drosophila* trachea to overcome these challenges. The *Drosophila* COPD model provides an excellent opportunity to systematically study the timeline of early and late structural damage, production of ROS, and inflammation upon pollutant exposure. Activity-based and phenotype-based drug screenings can be easily performed in the *Drosophila* COPD model, allowing for foundational studies that can provide valuable insight and background for future mammalian and human studies. In addition, elucidating the function of COPD-associated homologs and orthologs in the *Drosophila* trachea will provide evidence of the potential function of their human counterparts in COPD and allow for the development of novel and effective treatments. However, we should keep in mind that the data from studies in larval or pupal trachea would need to be verified in adult trachea as well, which is equivalent to human adult lungs.

## Figures and Tables

**Table 1:** COPD-associated human genes and *Drosophila* homolog/orthologs.

COPD-Associated Gene	Human Function	*Drosophila* Homolog/Ortholog
*ADAM19*	Cell matrix interactions	*Meltrin*
*ADRB2*	Β-2-adrenergic receptor	*Octβ2R*
*COL15A1*	Basement membrane adhesion to connective tissue	*Mp*
*HTR4*	Serotonin receptor	*Octβ1R*
*RAB4B*	Vesicular trafficking	*Rab4*
*FGF18*	Cell proliferation, differentiation, and migration	*bnl*
*SERP2*	Protect unfolded target proteins when under ER stress	*CG32276*
*RREB1*	Transcription factor that binds specifically to the RAS-responsive elements (RRE) of gene promoters	*peb*
*ID4*	Gene expression regulator of numerous cellular processes	*emc*
*TGFB2*	Tumor suppression, regulation of muscle and tissue development, wound healing, immune system function	*dpp* *daw*
